# Single‐cell and spatial alterations of neural cells and circuits in clinical and translational medicine

**DOI:** 10.1002/ctm2.1696

**Published:** 2024-05-29

**Authors:** William Wang, Xuanqi Liu, Diane Catherine Wang

**Affiliations:** ^1^ Office of Clinical and Translational Medicine Shanghai China; ^2^ Shanghai Institute of Clinical Bioinformatics Zhongshan Hospital Shanghai Medical College Fudan University Shanghai China; ^3^ Emergency Medicine, Sunshine Coast University Hospital Sunshine Coast Australia

**Keywords:** clinical, disease, medicine, single‐cell, translational

## Abstract

The spatiotemporal heterogeneity of neurons, circuits and regulators is being uncovered at a single‐cell level, from single‐cell gene expression to functional regulations. The classifications, architectonics and functional communications amongst neural cells and circuits within the brain can be clearly delineated using single‐cell multiomics and transomics. This Editorial highlights the spatiotemporal heterogeneity of neurons and circuits as well as regulators, initiates the translation of neuronal diversity and spatial organisation at single‐cell levels into clinical considerations, and enables the discovery and development of new therapies for neurological diseases. It is predicted that single‐cell and spatial multiomics will be integrated with metabolomic profiles and corresponding gene epigenetic modifications. The interactions amongst DNAs, RNAs and proteins in a cell provide details of intracellular functional regulations and new opportunities for the translation of temporospatial diversity of neural cell subtypes/states into clinical practice. The application of single‐cell multiomics with four‐dimensional genome to the human pathological brain will lead us to a new milestone of the diagnosis and treatment.

The complexity of the brain is highly dependent upon molecular and cellular diversities, functional and spatial regionalisation, and comprehensive and subtle network regulations. With the rapid development of biotechnologies, the single‐cell RNA sequencing (scRNA‐seq) and spatial transcriptome (ST) can provide new insights into understanding molecular and spatiotemporal profiles of cells in various organs and has the potential to greatly impact clinical and translational medicine.^1^, ^2^ In multiple organs, there is an increasing understanding on single‐cell transcriptomic and epigenetic census and atlas, chromatin accessibility and landscape, and molecular and cellular temporalisation and spatialisation. The NIH's Brain Research through Advancing Innovative Neurotechnologies (BRAIN) Initiative—Cell Census Network (BICCN) is a national program to establish an atlas of brain cell types and a census of the number and location of cells and neural circuits in a high‐resolution and definite spatialisation, correlated with neuronal morphology, connectivity, electrophysiology, and structures relevant for neurodegeneration and neuropsychiatric diseases (https://biccn.org). The BICCN demonstrated the molecular genetic landscape of cortical cell types and transcriptomes, chromatin and DNA methylation maps, diversities amongst species, spatial atlas of the motor cortex, and biological validity and genomic underpinning of neuron types.^3^, ^4^ The combination of scRNA‐seq with ST provides a multi‐dimensional and multi‐layered image of neuronal single‐cell types, organisation, and circuit function. As one of Clinical and Translational Medicine initiatives,^5^, ^6^, ^7^, ^8^, ^9^ clinical single‐cell biomedicine (cscBioMed) calls special attention to the scRNA‐seq/ST‐based trajectory of the neural circuit development, networks, regeneration, reconstruction, to discover neural spatiotemporal molecular imaging for new diagnostics, and to investigate the activation of target‐specific neurons and circuits for innovative therapies. This Editorial highlights the spatiotemporal heterogeneity of neurons, circuits, regulators, and the initiation of translation of neuronal diversity and spatial organisation at single‐cell levels into clinical considerations, and enables the discovery and development of new therapies for neurological diseases (Figure 1).

The neuron and circuit spatialisation and temporalisation reconstructed with scRNA‐seq/ST provides new insights into understanding and predicting the complexity of the human brain, although many challenges remain to drawing the full map of the brain. The landscapes of transcriptomics‐based neural clusters, cell‐types/subtypes/states, interneural connections, and heterogeneities have been investigated at the single‐cell resolution for more than 20 decades. With the rapid development of the single‐cell epigenomic approach, the combination of retrograde labelling with single‐nucleus DNA methylation sequencing (epi‐retro‐seq) illuminated cortical projection neuron types and degrees, their molecular and anatomical properties, and the connections between neurons.^10^ The artificial neural network model of mouse brain cell‐type/subtype epigenomes was established by integrating single‐nuclear DNA methylation sequencing, assay for transposase accessible chromatin with high‐throughput sequencing (ATAC‐seq), and epi‐retro‐seq.^4^ This particular model provides information on neural cell types/subtypes/states, definite spatialisations, projection specificities, epigenetic functions, gene expressions, and transcriptional regulatory networks and factors. It allows us to visualise single neuron functions and patterns of certain cell types, visualise spatialisations, and the functional and anatomical connections of neural circuits at a multi‐dimensional level. These are important proof that help us re‐consider molecular mechanisms of cortical projection neuron‐associated disorders, such as autism, depression, Alzheimer's disease, and Parkinson's disease.

The combination of scRNA‐seq with targeted functional measurements in the brain innovate new molecular mechanisms by which the differentiation and dynamics of neural spatialisation and inter‐communication are regulated and controlled. For example, the combination of scRNA‐seq with target cell labelling and mosaic functional analysis demonstrated that clustered protocadherins (cPCDHs), one of the cell adhesion molecules, orient and control the morphological spatialisation and functional organisation of clonally related excitatory neurons in the neocortex, where various neurons are constructed in multi‐layers and sophisticated circuits.^11^ In this particular study, the precise interneural recognising, arrangement developing, synaptic connecting and local constructing of excitatory neurons in the neocortex were regulated by the expression pattern and spatialisation of cPCDH genes. It indicates a new molecular mechanism that maintains the balance between excitation and inhibition in neocortical circuits and explain the pathogenesis of psychiatric disease states, risk genotypes, age, epileptogenic/antiepileptogenic repair, and neuropsychological and intellectual disorders (such as schizophrenia, bipolar disorder and autism). Spatial organisation and connectivity of various neural cell types/subtypes/states play decisive roles in the performance of the brain function and the formation of structural patterns. By combining scRNA‐seq and multiplexed error‐robust fluorescence in situ hybridisation (MERFISH), a comprehensive and high‐resolution transcriptomic and spatial cell‐type atlas for the whole adult mouse brain was discovered, including four nested levels of classification: 34 classes, 338 subclasses, 1201 supertypes and 5 322 clusters.^12^ The atlas contains neuronal and non‐neuronal cell types, including respectively 29 and 5 classes, 315 and 23 subclasses, 1 156 and 45 supertypes, and 5 205 and 117 clusters across the mouse brain. This is an initial exploration to figure out the multi‐dimensional connections amongst transcriptomic identities, spatial specificities, organisation diversities, and regionalisation‐specific structures and functions at single‐cell levels.

The importance of brain single‐cell sequencing in clinical and translational medicine has been emphasised since many years ago,^13^ with special focuses on experimental approaches, cell subpopulation identification, brain region diversity, and definition of specific brain cell types and functions. The major concerns were the accuracy of single‐cell identity marked gene panels and the molecular corresponding between brain single‐cell heterogeneity, function, development and diseases. Using scRNA‐seq, the characteristics and heterogeneous subpopulations of cells with the tumour microenvironment of gliomas were found to differ from those of lung‐to‐brain metastases.^14^ It provides evidence for potential clinical selection of target‐oriented therapies for primary brain cancer and secondary brain metastasis and explains the development of drug resistance or variable responses to therapies. Of distinct cancer‐associated fibroblast (CAF) subpopulations, the number and activities of antigen‐presenting CAFs was related with the metastasis of non‐small cell lung cancer to the bone through SPP1‐CD44 and SPP1‐PTGER4 pathways, while inflammatory CAFs with metastasis to the brain acted through the tyrosine kinase receptor mesenchymal‐epithelial transition factor and hepatocyte growth factor pathways.^15^ To understand pathogenesis of neurotropic virus infection in the brain, the spatial and cellular distribution of labelled virus and host cell responses were scanned with fluorescence micro‐optical sectioning tomography (fMOST) and scRNA‐seq.^16^ The infection routes were associated with three‐dimensional distribution of virus in experimental brain and interactions between infected neurons and macrophages.^17^ The infection severity was highly dependent upon the virus receptor distribution, binding affinity between virus and receptor, expression profiles of receptors or associated entry factors, cellular connectomes and regulomes, and fundamental cell–cell and gene–gene interactions.

More advances in scRNA‐seq‐based understanding of brain molecular and cellular structures and functions are urgently needed to enable translation to clinical application. One of the challenges is how to integrate and interpret the information of brain single molecular omics, multi‐omics, trans‐omics generated from experimental studies into the corresponding cells of human brain. The transcriptomic identities of brain cells need to be evaluated and matched with the corresponding protein expression and function, and the debate on the discrepancy between mRNA and protein expression and between protein expression and function, especially between transcriptomic and proteomic profiles still remains.^18^, ^19^ Even though there are correlations between mRNA and protein expression, it is important to ensure and translate the quantitative and qualitative measurements into clinical biochemistry. It is a challenge to illustrate the complex diversity and heterogeneity amongst cell subpopulations, spatialisations, interactions, neurotransmitter and neuropeptide co‐expression patterns in the brain. The Enhanced Electric Fluorescence in situ Hybridisation (EEL FISH) was proposed to ensure the quality and quantity of STs of human brain samples with a large panel of target genes, by electrophoretically transferring RNA to the capture surface and removing autofluorescent lipofuscin.^20^

Translational processes of neurological single‐cell temporalisation and spatialisation will be initiated from the evolution dynamics, neuron subtype functions, and epigenetic modifications. The single‐nucleus transcriptome was applied to map the evolution of the cerebellum from early neurogenesis to adulthood in humans, mice and the marsupial opossum, and found the developing trajectories and dynamics of early‐born subtypes in humans.^21^ The differentiation and changes of cell subtypes/states during the development were accompanied with single‐cell transcriptomic and spatial diversity which showed new expression trajectories and varied between species. A large number of cell‐subtypes/states‐specific genes and factors in regulations and intercellular communications during neuronal differentiation may be a new source to generate biology‐, function‐, and pathology‐specific biomarkers and targets for new diagnosis and therapy. The single‐cell and spatial transcriptomic profiling reveals that neuropeptide and BDNF signalling, MAPK and CREB activation, ubiquitination pathways, and synaptic connectivity in subtypes/states of neurons and astrocytes play important roles in the formation of long‐term memory,^22^ an initiation in single‐cell‐based memory investigations. Of neuron subtypes/states, neuron*^PENK+TAC−^* was discovered as the most important player in the memory engram of the basolateral amygdala to contribute to the long‐term memory by interacting with astrocytes. Memory‐specific transcriptomic profiles of neuron*^PENK+TAC−^* demonstrated potential mechanisms to orient the memory formation, regulate connections of the neural circuits and prevent brain aging. To explore gene expression and epigenetic regulation within neurons, the single nuclei in the tissue section were tagged with spatial barcode oligonucleotides from DNA‐barcoded beads with known positions (Slide‐tags) as a new strategy of single‐cell measurements in the ST.^23^ These tagged nuclei were integrated by single‐nucleus transcriptomic profiles with positioning and spatialisation of cell subtype‐specific transcriptomic identities open chromatins, and T cell receptors amongst neural cell layers and circuit networks for multimodal spatial genomics. The genomic distance‐dependent chromatic frequency was developed to further define the heterogeneity in the spatialisation of chromatin and RNA interactions. It aims to correlate the chromatin conformation with transcriptomic age in a cell of the human frontal cortex, as well as cell‐type‐specific functions with chromatin interactions, *Cis* expression quantitative trait loci with chromatin contacts, and links between XIST and chromosome X with structural variations in activations of X chromosome in female cortical cells.^24^ The introduction of single‐cell multiomics and transomics into the brain will provide new and deeper insights to understanding molecular mechanisms of neurological diseases and aging using multiple temporospatial dimensionalities constructed with transcriptomic expression, and interactions between chromatins and between RNA–chromatins in a cell.

In conclusion, the spatiotemporal heterogeneity of neurons and circuits as well as regulators is being uncovered at a single‐cell level, from single‐cell gene expression to functional regulations. The classifications, architectonics, and functional communications amongst neural cells and circuits within the brain are clearly delineated using single‐cell multiomics and transomics. It is expected that single‐cell and spatial multiomics can be integrated with metabolomic profiles and corresponding gene epigenetic modifications. The interactions amongst DNAs, RNAs, and proteins in a cell provide details of intracellular functional regulations and new opportunities for the translation of temporospatial diversity of neural cell subtypes/states into clinical considerations and for the discovery of new therapies for neurological diseases (Figure 1).

**FIGURE 1:**
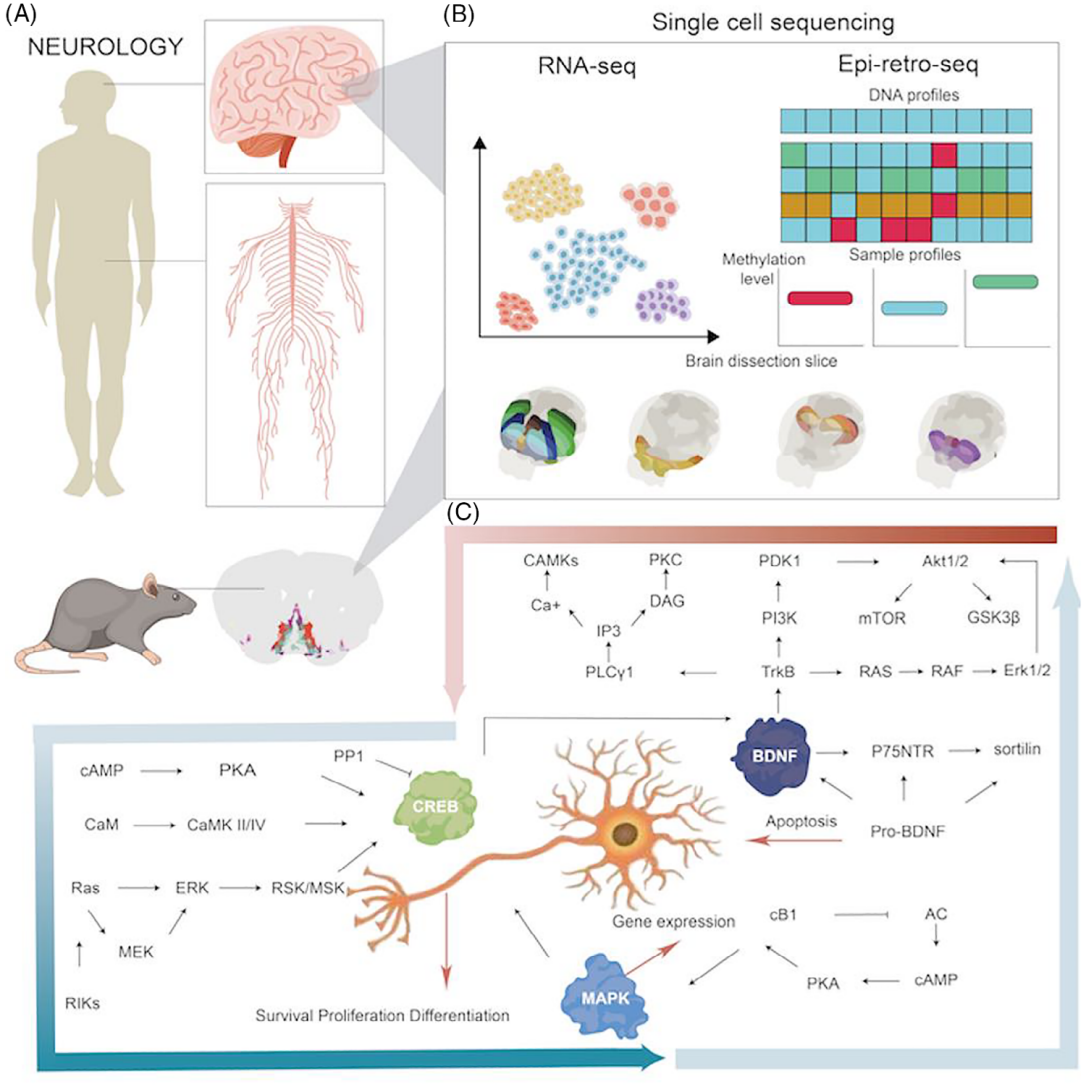
The application of single‐cell multi‐omics technologies in the field of nervous system. The human and mouse nervous systems are characterised by complex networks that make up the connectome, supporting the intricate cognition. The development, maintenance and function of the vertebrate nervous system depend on a sophisticated network of signalling pathways and are tightly regulated in spatialisation and temporalisation by various growth factors (A). In order to better understand the heterogeneity of neurons and regulatory factors in the nervous system. The cutting‐edge techniques like single‐cell multi‐omics analysis are applied. Through scRNA‐seq, epi‐retro‐seq, ATAC‐seq and spatial transcriptomics, the functional regulations and interactions of neurons and circuits are elucidated (B). The profiling of single‐cell and spatial transcriptomics reveals the crucial roles of neuropeptides, human brain‐derived neurotrophic factor (BDNF), mitogen‐activated protein kinase (MAPK), and cyclic‐AMP response element binding protein (CREB) signalling pathways in neurological diseases. The interactions between DNAs, RNAs, and proteins in the cell provide novel insights into intracellular regulation mechanism, offering new avenues for innovation in the clinical practice (C).

## AUTHOR CONTRIBUTIONS

Drs. Wang, Liu, and Wang contributed to the preparation and collection of original literatures and figures and the writing and editing of manuscript.

## CONFLICT OF INTEREST STATEMENT

The authors declare no conflicts of interest.

## ETHICAL APPROVAL

N/A

## Data Availability

Data sharing is not applicable to this Editorial as no new data were created or analysed in this study.

## References

[1] Zhang L , Chen D , Song D , et al. Clinical and translational values of spatial transcriptomics. Signal Transduct Target Ther. 2022;7(1):111. doi:10.1038/s41392-022-00960-w 35365599 PMC8972902

[2] Liu X , Jiang Y , Song D , et al. Clinical challenges of tissue preparation for spatial transcriptome. Clin Transl Med. 2022;12(1):e669. doi:10.1002/ctm2.669 35083877 PMC8792118

[3] BRAIN Initiative Cell Census Network (BICCN) , A multimodal cell census and atlas of the mammalian primary motor cortex. Nature. 2021;598(7879):86‐102. doi:10.1038/s41586-021-03950-0 34616075 PMC8494634

[4] Liu H , Zhou J , Tian W , et al. DNA methylation atlas of the mouse brain at single‐cell resolution. Nature. 2021;598(7879):120‐128. doi:10.1038/s41586-020-03182-8 34616061 PMC8494641

[5] Liu F , Liu X , Powell CA , et al. Initiative of clinical single‐cell biomedicine in clinical and translational medicine. Clin Transl Med. 2023;13(1):e1173. doi:10.1002/ctm2.1173 36629041 PMC9832424

[6] Wang X , Fan J . Spatiotemporal molecular imaging is a critical part of spatiotemporal molecular medicine. Clin Transl Med. 2021;11(3):e347. doi:10.1002/ctm2.347 33784006 PMC7933018

[7] Liu X , Wang DC , Powell CA , Wang X . Challenges of clinical translation from single‐cell sequencing to measures in clinical biochemistry of haematology: definition of immune cell identities. Clin Transl Med. 2023;13(9):e1401. doi:10.1002/ctm2.1401 37700496 PMC10497804

[8] Liu X , Powell CA , Wang X . Forward single‐cell sequencing into clinical application: understanding of cancer microenvironment at single‐cell solution. Clin Transl Med. 2022;12(4):e782. doi:10.1002/ctm2.782 35474615 PMC9042796

[9] Yan F , Li Z , Powell CA , Wang X . Forward single‐cell sequencing into clinical application: understanding of ageing and rejuvenation from clinical observation to single‐cell solution. Clin Transl Med. 2022;12(5):e827. doi:10.1002/ctm2.827 35593205 PMC9121316

[10] Zhang Z , Zhou J , Tan P , et al. Epigenomic diversity of cortical projection neurons in the mouse brain. Nature. 2021;598(7879):167‐173. doi:10.1038/s41586-021-03223-w 34616065 PMC8494636

[11] Lv X , Li S , Li J , et al. Patterned cPCDH expression regulates the fine organization of the neocortex. Nature. 2022;612(7940):503‐511. doi:10.1038/s41586-022-05495-2 36477535 PMC10249668

[12] Yao Z , van Velthoven CTJ , Kunst M , et al. A high‐resolution transcriptomic and spatial atlas of cell types in the whole mouse brain. Nature. 2023;624(7991):317‐332. doi:10.1038/s41586-023-06812-z 38092916 PMC10719114

[13] Duran RC , Wei H , Wu JQ . Single‐cell RNA‐sequencing of the brain. Clin Transl Med. 2017;6(1):20. doi:10.1186/s40169-017-0150-9 28597408 PMC5465230

[14] Sun HF , Li LD , Lao IW , et al. Single‐cell RNA sequencing reveals cellular and molecular reprograming landscape of gliomas and lung cancer brain metastases. Clin Transl Med. 2022;12(11):e1101. doi:10.1002/ctm2.1101 36336787 PMC9637666

[15] Xu K , Wang H , Zou YX , et al. Distinct fibroblast subpopulations associated with bone, brain or intrapulmonary metastasis in advanced non‐small‐cell lung cancer. Clin Transl Med. 2024;14(3):e1605. doi:10.1002/ctm2.1605 38445456 PMC10915739

[16] Zhang Y , Xing X , Long B , et al. A spatial and cellular distribution of rabies virus infection in the mouse brain revealed by fMOST and single‐cell RNA sequencing. Clin Transl Med. 2022;12(1):e700. doi:10.1002/ctm2.700 35051311 PMC8776042

[17] Chen D , Ou Z , Zhu J , et al. Screening of cell–virus, cell–cell, gene–gene crosstalk among animal kingdom at single cell resolution. Clin Transl Med. 2022;12(8):e886. doi:10.1002/ctm2.886 35917402 PMC9345398

[18] Li J , Zhang Y , Yang C , Rong R . Discrepant mRNA and protein expression in immune cells. Curr Genomics. 2020;21(8):560‐563. doi:10.2174/1389202921999200716103758 33414677 PMC7770634

[19] Buccitelli C , Selbach M . mRNAs, proteins and the emerging principles of gene expression control. Nat Rev Genet. 2020;21(10):630‐644. doi:10.1038/s41576-020-0258-4 32709985

[20] Borm LE , Mossi Albiach A , Mannens CCA , et al. Scalable in situ single‐cell profiling by electrophoretic capture of mRNA using EEL FISH. Nat Biotechnol. 2023;41(2):222‐231. doi:10.1038/s41587-022-01455-3 36138169 PMC9931581

[21] Sepp M , Leiss K , Murat F , et al. Cellular development and evolution of the mammalian cerebellum. Nature. 2024;625(7996):788‐796. doi:10.1038/s41586-023-06884-x 38029793 PMC10808058

[22] Sun W , Liu Z , Jiang X , et al. Spatial transcriptomics reveal neuron‐astrocyte synergy in long‐term memory. Nature. 2024;627(8003):374‐381. doi:10.1038/s41586-023-07011-6 38326616 PMC10937396

[23] Russell AJC , Weir JA , Nadaf NM , et al. Slide‐tags enables single‐nucleus barcoding for multimodal spatial genomics. Nature. 2024;625(7993):101‐109. doi:10.1038/s41586-023-06837-4 38093010 PMC10764288

[24] Wen X , Luo Z , Zhao W , et al. Single‐cell multiplex chromatin and RNA interactions in ageing human brain. Nature. 2024. doi:10.1038/s41586-024-07239-w PMC11023937 38538789

